# Review: transcriptome and trans-omics analysis of systemic lupus erythematosus

**DOI:** 10.1186/s41232-020-00123-w

**Published:** 2020-06-18

**Authors:** Keishi Fujio, Yusuke Takeshima, Masahiro Nakano, Yukiko Iwasaki

**Affiliations:** Department of Allergy and Rheumatology, Graduate School of Medicine, The University of Tokyo, 113-8655 7-3-1 Hongo, Bunkyo-ku, Tokyo, Japan

**Keywords:** Systemic lupus erythematosus, Transcriptome, Omics, eQTL

## Abstract

Systemic lupus erythematosus (SLE), which was recognized as a defined clinical entity more than 100 years ago, is an archetype for systemic autoimmune diseases. The 10-year survival of SLE patients has shown dramatic improvement during the last half-century. However, SLE patients receiving long-term prednisone therapy are at high risk of morbidity due to organ damage. Identification of key immune pathways is mandatory to develop a suitable therapy and to stratify patients based on their responses to therapy. Recently developed transcriptome and omic analyses have revealed a number of immune pathways associated with systemic autoimmunity. In addition to type I interferon, plasmablast and neutrophil signatures demonstrate associations with the SLE phenotype. Systematic investigations of these findings enable us to understand and stratify SLE according to the clinical and immunological features.

## Background

Systemic lupus erythematosus (SLE) is an archetype of autoimmune disease. It is characterized by its predominance in woman of childbearing age [1, 2]. Clinical heterogeneity, together with the potential severity of these manifestations, make the management of SLE a distinct challenge for rheumatologists. Despite recent progress in treatment protocols, several studies have pointed out that SLE patients still have an overall 2- to 3-fold increased risk of death [3, 4]. In this context, an initiative to evaluate possible therapeutic targets and develop *treat-to-target* guidance would be highly appropriate in the management of SLE patients.

The Asia Pacific Lupus Collaboration (APLC) proposed the existence of a Lupus Low Disease Activity State (LLDAS). This was subsequently defined operationally via a nominal consensus approach [5]. LLDAS was defined as follows: SLE Disease Activity Index (SLEDAI) 2K ≦ 4, with no activity in major organ systems; no new lupus disease activity compared with the previous assessment; SLEDAI Physician Global Assessment 1 ≦ 4; a current prednisolone (or equivalent) dose ≦ 7.5 mg daily; and well-tolerated standard maintenance doses of immunosuppressive drugs. Patients who spent greater than 50% of their observed time in LLDAS had significantly reduced accrual of organ damage compared with patients who spent less than 50% of their time in LLDAS and were significantly less likely to have increased organ damage. However, some patients developed organ damage in spite of the maintenance of LLDAS. While some patients remain serologically active clinically quiescent (SACQ) indefinitely or become serologically quiescent clinically quiescent (SQCQ), others’ SACQ periods are terminated by disease flares for which reliable predictors have yet to be identified [6]. Therefore, novel biomarkers are necessary to improve prognostic stratification, monitoring of treatment responses, and detection of early renal flares. Transcriptome and omics analyses, which evaluate the genome, transcriptome, and epigenome, are powerful approaches to the identification of clinically useful biomarkers.

## Key molecular signatures in SLE

SLE patients demonstrate transcriptional signatures related to type I interferon (IFN) and granulocytes [7, 8]. Although a number of studies have focused on the linkage between IFN-induced transcripts or proteins and SLE disease activity, most of them suffer from sample-size limitations and essential clinical and therapeutic heterogeneity in SLE. Chaussabel et al. applied modular analysis in PBMC microarray data from 239 individuals [9]. Differentially expressed gene (DEG) analysis tolerates noise due to the large number of comparisons performed (usually > 10,000). In order to reduce noise, they focused the analysis on small sets of coordinately expressed transcripts that constitute coherent and biologically meaningful transcriptional units. Among 28 transcriptional modules linked with pathways or cell types involved in immune processes, two modules, IFN-inducible and neutrophil genes, exhibited positive correlations with SLE disease activity, SLEDAI. In contrast, gene modules corresponding to ribosomal proteins and surface molecule of T cells including CD5, CD6, CD7, CD26, CD28, and CD96 negatively correlated with SLEDAI. Intriguingly, in one patient who showed disease flaring and subsequent recovery, an increase in the transcriptional score was detected 2 months before deterioration of the patient’s clinical condition. Their following report also revealed complex IFN signatures in SLE that were composed of 3 modules and involved both an IFN-α signature and those for IFN-β and IFN-γ [10].

The possibility of clinical application of IFN gene expression data has been demonstrated by a two-score system for IFN status. Factor analysis by El-Sherbiny et al. in 328 autoimmune disease patients reduced the 30 ISGs (interferon signature genes) to 2 factors, IFN-Score-A and IFN-Score-B [11]. Although IFN-Score-A and IFN-Score-B shared several key ISGs, specific genes for IFN-Score-A were mostly typical ISGs, i.e., *IFIT1*, *IFI27*, *RSAD2*, *IFI44L*, *IRF7*, *ISG15*, and *XAF1*. On the other hand, specific genes for IFN-Score-B were *LAMP3*, *UNC93B1*, *SP100*, *IFI16*, *IFIH1*, *PHF11*, and *BST2*. IFN-Score-A differentiated SLE from both rheumatoid arthritis (RA) and healthy controls (HC), while IFN-Score-B differentiated SLE and RA from HC. In SLE, both scores were associated with cutaneous and hematological but not musculoskeletal disease activity. Yusof et al. examined these scores in 118 individuals at risk of autoimmune connective tissue diseases [12]. At baseline, both IFN scores were elevated in those at-risk who progressed to connective tissue diseases at 12 months versus nonprogressors, to a greater extent for IFN-Score-B (3.22-fold difference) than IFN-Score-A (2.94-fold difference). The fold-difference between at-risk and HCs for IFN-Score-A was markedly greater in the skin than blood. These results suggested potential application of IFN-score in lupus patients.

Banchereau et al. reported a longitudinal modular study of 158 SLE patients in the Immunological Genome Project Consortium [13]. They found that positive correlates of disease activity were enriched for the IFN response as well as plasmablast, cell cycle, neutrophil, histone, and B cell modules. Notably, the plasmablast signature was the most reproducible, and plasmablast responses showed increases in African-American patients who presented with more severe disease than did Caucasians. In addition, the neutrophil, myeloid lineage, and inflammation modules were enriched in patients with lupus nephritis. These observations support a model of sequential disease progression, whereby an initial increase in IFN response and differentiation of B cells into short-lived plasmablasts, which may occur before clinical onset, is followed by lupus nephritis and full-blown systemic inflammation enhanced by myeloid cells, including neutrophils.

One limitation of transcriptome analysis of peripheral blood cells or PBMC is the distorting effects of variations in the size of each immune subset. That is, transcriptome analyses of a mixture of cell subsets are obscured by the variations in the frequencies of different cell types, as shown in comprehensive analyses in the human brain of bulk/single-cell transcriptomes, active enhancers, genotypes, and Hi-C data sets, named PsychENCODE [14]. In PsychENCODE, weighted combinations of single-cell signatures could account for most of the population-level expression variation, with an accuracy of > 88%. Therefore, more detailed analyses of immune cell subsets are required to advance modular findings obtained from peripheral blood or PBMC. McKinney et al. conducted a subset-specific transcriptomic analysis of 58 patients with ANCA-associated vasculitis (AAV) and 23 patients with SLE. They found that while CD4^+^ T cell co-stimulation is pronounced, genes specifically downregulated in exhausted CD8^+^ T cells during chronic murine lymphocytic choriomeningitis virus (LCMV) infection were downregulated in CD8^+^ T cells from patients at low risk of relapse of AAV and SLE. This result suggested that whereas CD8^+^ exhaustion is associated with poor outcome in viral infection, it predicts favorable outcome in autoimmune diseases.

Shi et al. studied monocytes from 9 SLE patients using RNA-seq of both coding and noncoding RNAs [15]. They found that SLE patients had diminished expression of most endogenous retroviruses and small nucleolar RNAs. Conversely, lupus monocytes exhibited increased expression of pri-miRNAs and altered splicing patterns and polyadenylation. Rai et al. analyzed peripheral blood RNA-seq data from 28 SLE patients [16] to find that anti-dsDNA-positive patients showed dysregulation of multiple cytokine signaling pathways. In contrast, anti-extractable nuclear antigen (ENA)-positive patients exhibited specific dysregulation of IFN signaling.

### Approach to lupus nephritis

*Lupus nephritis* is a major risk factor for overall morbidity and mortality in SLE. Kidney biopsy and histological evaluation is the gold standard to determine the optimal treatment protocol. Prediction of prognosis based upon histological classification is insufficient, and transcriptomic information may improve patients’ stratification. Parikh et al. examined the expression of 511 immune-response genes with NanoString in kidney biopsies from 19 patients [17]. The top genes responsible for clinical response included several interferon pathway genes (*STAT1*, *IRF1*, *IRF7*, *MX1*, *STAT2*, *JAK2*), while complement genes (*C1R*, *C1QB*, *C6*, *C9*, *C5*, *MASP2*) were mainly responsible for non-clinical response.

Single-cell RNA-seq is a powerful approach that enables investigators to comprehensively evaluate inflamed organs at single-cell resolution [18, 19]. Based on the transcriptional signatures of such immune cells, the data reveal the immunological nature of inflammation and novel biomarkers. The Accelerating Medicines Partnership (AMP) is a public-private partnership created to develop new ways of identifying and validating promising biological targets for diagnostics and drug development. Arazi et al. analyzed kidney samples from 24 patients with lupus nephritis and from 10 control subjects using single-cell RNA sequencing [20]. They identified 21 subsets of leukocytes in lupus nephritis, including multiple populations of myeloid cells, T cells, natural killer cells, and B cells that demonstrated both pro-inflammatory responses and inflammation-resolving responses. Similar to peripheral blood cells, almost all immune cell subsets in the kidney demonstrated elevation of type I interferon. In particular, local activation of B cells was consistent with an age-associated B cell (ABC) signature that contributes to cytokine production and antigen presentation [21]. Trajectory analysis identifies a continuum of intermediate states spanning patrolling, phagocytic, and alternatively activated monocytes, suggesting progressive stages of monocyte differentiation within the kidney.

Der et al. applied single-cell RNA-seq to kidney and skin biopsy samples from 16 lupus nephritis patients [22]. IFN signatures in tubular cells and keratinocytes were specific features of patients with LN. Moreover, a high IFN signature and extracellular matrix (ECM) fibrotic signature in tubular cells were each associated with resistance to therapy. When they proposed an equation predicting response to treatment at 6 months post-biopsy, four genes, *COL1A1*, *COL14A1*, *COL1A2*, and *COL5A2*, were found to significantly explain variance and predict response to treatment with a 92% accuracy. With greater sample sizes, their analysis may help to identify disease-relevant or disease subtype-related populations that only exist in affected organs.

### Combining transcriptomics and genetic analysis

Genome-wide association study (GWAS) revealed a number of single nucleotide polymorphisms (SNPs) and genes associated with SLE pathogenesis [23]. GWAS has successfully identified more than 90 SLE risk loci that explain a significant proportion of SLE’s heritability. GWAS-SNP genes suggest several pathogenic pathways in SLE such as cytokine pathways including immune complex processing and phagocytosis, DNA degradation, neutrophil, and monocytes signaling; Toll-like receptor and/or type I interferon signaling; nuclear factor kappa beta (NF-κB) activation; and B and T cell function and signaling [24–26]. However, one of the great challenges posed by interpreting GWAS data is determining the causal genes implicated by the genetic association data. The association of a locus with disease does not specify which gene is affected by the causal variant. The fact that > 90% of disease-associated variants are located in non-protein-coding regions of the genome suggests that many loci implicated by GWAS alter the genetic regulation of one or more target genes. Therefore, combinatory analysis using both transcriptomics and genetic analysis may precisely reveal target genes for disease-related SNP and detailed immunological pathogenesis.

Zhang et al. simultaneously obtained RNA-seq and H3K4me3 Chip-seq data in T cells, B cells, and monocytes from 6 SLE patients [27]. Across the 3 cell types, there was 55% concordance for gene expression changes related to SLE. Key conserved pathways were ribosome biogenesis among upregulated genes and heat shock response among downregulated genes. Upon exploration of a common pathway linking H3K4me3 and gene expression, ATF3 showed a significant association across all cell types in both RNA-seq and ChIP-seq data sets. ATF3 is inducible by type I interferons in macrophages, providing a potential mechanism for increased function in SLE. When they compared the change in expression and change in H3K4me3 near the GWAS variants, there was a decrease in RNA abundance in all three cell types near the GWAS variants. With regard to H3K4me3, monocytes exhibited decreased H3K4me3 in GWAS variant region. These data are consistent in supporting the concept that GWAS variants impact gene regulation.

Genetic and epigenetic fine mapping has shown that most of the immune-mediated disease (IMD)-related SNPs are located within enhancer regions [28]. As enhancers regulate expression of target genes, SNPs in enhancer regions affect the expression levels of target genes. This effect, called expression quantitative trait locus (eQTL), provides important information regarding the function and role of disease-related SNPs and genes [29]. Bentham et al. performed large-scale GWAS comprised 7219 cases and 15,991 controls of European ancestry, combined with eQTL analysis using monocytes and B cells transcriptome data from database [23]. They identified eQTL effects and candidate causal genes in each immune cell subset and found an over-representation of 16 transcription factors among SLE susceptibility genes. However, there was a limitation that transcriptome data was not derived from SLE patients. Panousis et al. combined genotype and RNA-sequencing data to comprehensively evaluate blood transcriptomes in 142 SLE patients [30]. As anticipated, principal component analysis (PCA) discriminated transcriptomes in clinically active, inactive patients, and healthy individuals. On the other hand, inactive SLE cases were differentiated from healthy but not from active SLE, denoting persistently deregulated gene expression despite remission. Therefore, they determined a “susceptibility” signature that persists in the absence of disease activity following treatment. These genes were enriched in *regulation and response of the immune system* processes suggesting the persistence of immune activation. Notably, a total of 365 DEGs from the inactive versus active SLE comparison that are not in the “susceptibility” signature were enriched for *oxidative phosphorylation*, *ribosomes*, and *cell cycle*. With regard to organ damage, neutrophil activation and humoral response signatures correlated with lupus nephritis. Furthermore, Panousis et al. evaluated by eQTL analysis the extent to which transcriptomic changes in SLE may be genetically determined. eQTL analysis revealed 9 genes where both GWAS and eQTL identified the same variant, namely *UBE2L3*, *HLA-DRB5*, *RP11-356I2.3*, *BLK*, *FAM167A*, *NADSYN1*, *RP11-660 L16.2*, *ALDH2*, and *ALDH18A1*, thus implicating them in SLE pathogenesis.

Cytokines play central roles in the network of systemic autoimmunity. Davenport et al. examined interactions between eQTLs and cytokine levels in a lupus clinical trial to define target genes and mechanisms [31]. They conducted whole-blood RNA-seq in subjects who had or had not been treated with anti-IL6 at 0, 12, and 24 weeks of treatment. eQTL analysis of 379 samples from 157 patients revealed 4818 cis eQTL genes. When they classified each sample as either IFN high or IFN low using real-time PCR of 11 IFN-inducible genes, they identified 210 eQTLs which effect is magnified in IFN-high samples. Similarly, they identified 121 eQTLs which effect is magnified under anit-IL-6 drug treatment. Biologically relevant anti-IL-6 drug-eQTL interactions were noted for IL10, an anti-inflammatory cytokine, *CLEC4C*, which has previously been associated in trans with an SLE risk allele, and *CLEC18A*, another member of the C-type lectin domain family. Notably, the IRF4 motif was enriched among eQTLs which showed enhanced effect under anti-IL-6 drug treatment, suggesting that the interruption of transcription factor binding motifs by eQTL interaction SNPs may be a key regulatory mediator of environmental stimuli and potential therapeutic targets.

### T cell receptor and B cell receptor repertoires

Autoreactive T cells play critical roles in organ inflammation and promotion of autoantibody development. T cell receptors (TCR) that recognize autoantigens are generated by genomic rearrangement of the variable (V), diversity (D), and joining (J) regions. Expansion of the partial TCRs and reduction of TCR repertoire diversity were observed in peripheral blood cells of patients diagnosed with autoimmune disease [32]. RNA-seq effectively reveals the detailed nature of the TCR repertoire. Liu et al. examined the TCR repertoire using RNA-seq in peripheral blood cells obtained from 877 SLE patients, 206 RA patients, and 439 HCs [33]. Significant differences were identified in V, J, and V-J pairing among the SLE or RA and HC groups. Intriguingly, the 3 groups were almost perfectly discriminated by these differences. Consistent with previous studies reporting the importance and antigenic association of public TCRs in various diseases, TCR clones with a higher publicity in SLE and RA had higher clonal frequencies compared with HCs. They identified 198 SLE-associated TCR clones and 53 RA-associated clones. Moreover, in SLE, the Gini index of the V-J gene combinations and the content of SLE-associated clones were positively correlated with *SLEDAI*. The content of SLE-associated TCR clones and the total unique clone number were correlated with complement C3 and C4.

As is the case with the TCR, understanding of the BCR repertoire in the context of IMD could provide us new insights into pathogenesis and therapy. Bashford-Rogers et al. compared the BCR repertoire in SLE, AAV, Crohn’s disease, Behçet’s disease, eosinophilic granulomatosis with polyangiitis, and immunoglobulin A (IgA) vasculitis [34]. The BCR repertoire of SLE showed an increase in clonality and a dominance of the IgA isotype, suggesting a microbial contribution to disease. When the effect of immunosuppressive therapy was evaluated, MMF (mycophenolate mofetil) increased the proportion of IgM^+^ and IgD^+^ B cells and reduced the number of isotype-switched B cells. Conversely, after rituximab treatment in SLE, the number of circulating B cells was low but persisting cells were largely isotype-switched and clonally expanded IgG1- or IgG2-expressing cells. These characteristics of the TCR and BCR repertoire, particularly the disease-associated clones, may become useful biomarkers.

## Conclusion

Transcriptomic and omics analyses have revealed several important features of immunity in SLE. (1) IFN-inducible, neutrophil, and plasmablast genes positively correlate with SLE disease activity and lupus nephritis. (2) IFN scores predict the development of systemic autoimmune diseases. (3) CD8 exhaustion predicts favorable outcome in autoimmune diseases. (4) B cells with an ABC phenotype infiltrate and monocytes progressively differentiate in lupus kidneys. ECM fibrotic signature in tubular cells is associated with resistance to therapy. (5) Public TCR in SLE is highly disease-specific, and the BCR repertoire of SLE showed an increase in clonality and dominance of an IgA isotype. This finding will help us to understand the pathogenesis of lupus and to stratify essentially heterogenous SLE patients.

## Data Availability

Not applicable

## Abbreviation

SLE: Systemic lupus erythematosus
LLDAS: Lupus Low Disease Activity State
IFN: Interferon
DEG: Differentially expressed gene
SACQ: Serologically active clinically quiescent
SLEDAI: SLE Disease Activity Index
AAV: ANCA-associated vasculitis
GWAS: Genome-wide association study
SNPs: Single nucleotide polymorphisms
IMD: Immune-mediated disease
eQTL: Expression quantitative trait locus
PCA: Principal component analysis
TCR: T cell receptors
